# Controlled ovarian hyperstimulation induced changes in the expression of circulatory miRNA in bovine follicular fluid and blood plasma

**DOI:** 10.1186/s13048-015-0208-5

**Published:** 2015-12-09

**Authors:** Sina Seifi Noferesti, Md. Mahmodul Hasan Sohel, Michael Hoelker, Dessie Salilew-Wondim, Ernst Tholen, Christian Looft, Franca Rings, Christiane Neuhoff, Karl Schellander, Dawit Tesfaye

**Affiliations:** Animal Breeding and Husbandry Group, Institute of Animal Science, University of Bonn, Bonn, 53115 Germany; Department of Animal Science, Faculty of Agriculture, Erciyes University, Kayseri, 38039 Turkey

**Keywords:** Extracellular miRNA, Ovarian hyperstimulation, Follicular fluid, Exosome, Ago2

## Abstract

**Background:**

Despite its role in increasing the number of offspring during the lifetime of an individual animal, controlled ovarian hyperstimulation (COH) may have detrimental effects on oocyte development, embryo quality and endometrial receptivity. Circulating miRNAs in bio-fluids have been shown to be associated with various pathological conditions including cancers. Here we aimed to investigate the effect of COH on the level of extracellular miRNAs in bovine follicular fluid and blood plasma and elucidate their mode of circulation and potential molecular mechanisms to be affected in the reproductive tract.

**Method:**

Twelve simmental heifers were estrous synchronized and six of them were hyperstimulated using FSH. Follicular fluid samples from experimental animals were collected using ovum pick up technique at day 0 of the estrous cycle and blood samples were collected at day 0, 3 and 7 of post ovulation. The expression profile of circulatory miRNAs in follicular fluid and blood plasma were performed using the human miRCURY LNA™ Universal RT miRNA PCR array system. A comparative threshold cycle method was used to determine the relative abundance of the miRNAs.

**Results:**

A total of 504 and 402 miRNAs were detected in both bovine follicular fluid and blood plasma, respectively. Of these 57 and 21 miRNAs were found to be differentially expressed in follicular fluid and blood plasma, respectively derived from hyperstimulated versus unstimulated heifers. Bioinformatics analysis of those circulating miRNAs indicated that their potential target genes are involved in several pathways including TGF-beta signaling pathway, MAPK signaling pathway, pathways in cancer and Oocyte meiosis.

Moreover, detail analysis of the mode of circulation of some candidates showed that most of the miRNA were found to be detected in both exosomal and Ago2 protein complex fraction of both follicular fluid and blood plasma.

**Conclusion:**

Our data provide the consequence of hyperstimulation induced changes of extracellular miRNAs in bovine follicular fluid and blood plasma, which may have a potential role in regulating genes associated not only with bovine ovarian function but also involved in altering various physiological in bovine oocytes, embryos and modulating reproductive tract environment.

## Background

Follicular development is a dynamic process which involves emergence of a group of follicles after an increase of peripheral concentration of follicle stimulating hormone (FSH) [1]. Follicular products from the dominant follicle are responsible for suppressing FSH concentration and leading to atresia of the subordinate follicles [2, 3]. Meanwhile, dominant follicle acquires LH receptors in its granulosa cell and no longer dependent of FSH for further development. Maintenance of elevated level of circulating FSH rescues the subordinate follicles from regression, resulted in the growth of multiple dominant follicles with ovulating capability [4]. Therefore, Controlled ovarian hyperstimulation (COH) is utilized to stimulate the growth of multiple follicles in naturally mono-ovulating species, including cattle and humans, by maintaining supraphysiological level of gonadotrophin.

Although COH is the central component of ART, it is recently became evident that COH may have detrimental effects on oocyte development, embryo quality, endometrial receptivity and perhaps on perinatal outcomes [5]. In vitro studies showed that ovarian stimulation disrupts and delays early embryonic development in mouse [6, 7]. We have previously shown that changes in the bovine oviduct and uterine environment induced by gonadotrophin treatments has resulted in changes in gene expression profile of the resulting blastocysts [8]. Furthermore, an increase in chromosomal abnormalities has also been reported in human embryos after conventional stimulation, mainly resulting from an increased incidence of chromosome segregation errors during the first embryonic divisions [9]. It is well known that COH leads to deviant oocyte development in the follicle [10] as a result of nonphysiological endocrine level in the reproductive organs. Follicular development is a complex coordinated process facilitated by the bidirectional signalling between gonadal and somatic cells in follicular microenvironment. A large number of gene products are involved in this process, which are expressed in cell type and time dependent manner Any deviation in the expression of these genes due to environmental factors could lead to abnormal oocyte development, apoptosis, and poor cellular communication with the companion somatic cells [11]. In this context the role of miRNAs as post transcriptional regulaters of gene expression can not be ruled out as these regulatory molecules are abundantly expressed in follicular cells and in extracellular space in follicular microenvironment. MicroRNAs (miRNAs) are a class of small non-coding RNAs that regulate gene expression at the post-transcriptional level. miRNAs have been found in all mammalian tissues and cell types examined so far and play important roles in a variety of physiological and pathological processes [12]. While majority of miRNAs are detected intracellular, a handful number of miRNAs are also detected in extracellular body fluids such as plasma, serum, urine, saliva and follicular fluid [13–15]. Although the origin of cell-free miRNAs in circulation is not entirely clear, miRNAs are believed to be released to the extracellular space from damaged or apoptotic cells and are also actively secreted by cells via exosomes or exocytosis [13]. Circulating miRNAs have been shown to be useful biomarkers for multiple clinical endpoints including the diagnosis of preeclampsia [16], the diagnosis and monitoring of diabetic retinopathy [17], neuropathy [18] and as diagnostic or prognostic markers for multiple cancers [14, 19–22]. Cellular release of miRNA molecules into the circulation has been shown to be associated with the altered physio-pathological conditions. The function of exosomes and shedding vesicles are believed to be cell-to-cell communication and platforms for multi signalling processes [23, 24].

Circulating miRNAs have been extensively studied since their discovery [13, 14], and they are known to be coupled with vesicles, in particular microvesicles or exosomes [25] or high density lipoproteins (HDL) [24, 26, 27] and protein complexes [28–30] and their presence in the blood has been attributed to release by tissue injury. We have recently demonstrated that the presence of extracellular miRNAs in bovine follicular fluid and their association with the growth status of oocytes [15]. However, effect of gonadotrophin treatments during COH on extracellular miRNAs in follicular fluid and blood plasma has not been investigated yet in mammalian species. Therefore, the aim of this study was to test the hypotheses that controlled ovarian hyperstimulation may change the extracellular miRNA population in follicular fluid and blood plasma of cyclic heifers, which may be involved in various physiological processes. For this a qRT-PCR array based expression profiling was used to identify a panel of differentially expressed circulating miRNAs in follicular fluid samples and blood plasma samples of hyperstimulated compared to unstimulated heifers. Moreover, the bioinformatics analysis of the candidate miRNAs revealed the potential involvement of those miRNAs in regulation of several signaling pathways, which are known to be active in various key reproductive processes.

## Methods

### Animal treatments and sample collection

All experimental animals were handled according to the animal protection law of Germany. The experiment was approved by the Animal Welfare committee of the University of Bonn with proposition number 84–02.05.20.12.075. Simmental heifers (*n* =10), aged from 15 to 17 months and weighing between 380 to 450 kg were used in this study. All animals were kept under identical farm conditions within the same herd. Synchronization and ovarian hyperstimulation was performed according to the previously mentioned protocol [8] Briefly, pre-synchronization was performed for all animals by intra-muscular administration of 500 mg of cloprostenol (PGF2a, Estrumatew; Essex Tierarznei, Munich, Germany) twice within 11 days. Two days after each of the PGF2a treatments animals received 10 mg of GnRH (Receptalw; Intervet, Boxmeer, the Netherlands). Of 10 synchronized heifers 6 were used for hyperstimulation in which twelve days after the last GnRH injection, these heifers received the first of eight consecutive FSH-injections over 4 days in decreasing doses (in total 300–400 mg of FSH equivalent according to the body weight; Stimufol, University of Liege, Belgium). Two PGF2a treatments were performed 60 and 72 h after the initial FSH injection. Finally, 48 h after the application of first PGF2a, ovulation was induced by simultaneous administration of 10 mg of GnRH. Afer 60 h of first PGF2a application was considered as onset of oestrus (D0). Follicular contents (follicle 35 mm) were collected by transvaginal, ultrasound-guided follicular aspirations. Follicular fluid was collected using a 12-gauge needle, centrifuged at 1500 × g for 5 min, and later stored at −80 °C, while blood samples were collected from each animal from day 0 (D0), day 3 (D3) and day7 (D7) by tail vein puncture. Blood serum following collection, blood samples were refrigerated at 4 °C for 12–24 h before being centrifuged at 1500 × g at 4 °C for 15 min. Serum was separated and stored at −20 °C until assayed to determine progesterone concentration. Blood plasma for miRNA detection was collected by EDTA Tubes (Carl Roth, Karlsruhe, Germany) from the both group animals and stored at −80 °C until processed for microvesicles/ exosomes, RNA, or protein isolation.

### Progesterone assay

Serum progesterone concentration in different time points was determined by time-resolved immunofluorescence using an Auto DELFIA™ Progesterone kit (Perkin Elmer, Wallac Oy, Turku, Finland) which is based on the fluorescence of elements where the assay sensitivity was 0.01 ng/ml. The assay principle combines an enzyme immunoassay competition method with final fluorescent detection. The DELFI test is based on the competition for binding sites on the antibody molecule that occurs between the Europium + 3-labeled hormone and a not-labeled hormone, contained in the sample. The amount of the labeled hormone is constant, whilst the not-labeled hormone content is a function of antibody- labeled hormone complex formation. On this basis, a standard curve was drawn for reading the hormone levels in the sample.

### Isolation total RNA and reverse transcription

Total RNA was isolated from follicular fluid and blood plasma, ultracentrifugation pellets and immunoprecipitation pellets using the miRNeasy kit (Qiagen, Hilden, Germany) according to the manufacturer’s protocol with some modifications. Briefly, 800 μL of QIAzol buffer was added to 200 μL of plasma or follicular fluid or exosome pellet or Ago2 pellet and incubated at room temperature for 8 min. After that to inactivate RNases activity 200 μL chloroform was added to each sample. At that point, the manufacturer’s protocol was followed. Total RNA concentration and purity was determined using NanoDrop ND-1000 spectrophotometer. Moreover, prior to reverse transcription procedure RNA samples from both plasma and follicular fluid were checked for the presence or absence of PCR inhibitors described elsewhere [15]. Briefly, cDNA was synthesized using different input volume, for instant 0.5 μl, 1 μl, 2 μl and 4 μl of total RNA, in 10 μl of reaction volume and quantify the expression of selected miRNA using qPCR. All RNA samples showed linear amplification of the candidate miRNAs for different levels of RNA input, which showed the absence of PCR inhibitors and good quality RNA (data not shown). A reverse transcription reaction was performed using the miRCURY LNA™ Universal RT microRNA PCR system (Exiqon, Denmark) according to the manufacturer’s instructions. In brief, approximately a total of 100 ng of total RNA, including small RNA, were anchor-tailed with a poly(A) sequence at their 3'end and then reverse transcribed into cDNA using a universal poly(T) primer with a 3'end degenerate anchor and a 5' end universal tagged.

### miRNA Profiling and expression analysis

The expression of miRNAs in blood plasma and follicular fluid were profiled using miRNA Ready-to-Use PCR, Human Panel I &II, V2.M qRT-PCR arrays (Exiqon, Vedbaek, Denmark) which contains 748 mature miRNA sequences along with 8 empty wells, 6 intra-plate calibrators and 6 endogenous controls. A SYBR green based real time PCR technology was employed for signal detection during quantification. Prior to real time PCR analysis the cDNA products from each samples were diluted 100-fold and mixed with ready to use SYBR-green master mix. Then the cDNA-master mix was robotically pipette to a 384-well PCR plate containing miRNA specific primers. The real time PCR was run on a ABI-7900HT thermocycler (Applied Biosystems) using the following thermal-cycling parameters: 95 °C for 10 min, 40 cycle of 95 °C for 10 sec, 60 °C for 1 min followed by a melting curve analysis. Raw Ct values were calculated as recommended by Exiqon using the RQ manager software v1.2.1 (Applied Biosystems), and Ct values were calculated by using automated assay-specific baseline and threshold settings. To minimize the potential noise, miRNAs with Ct value greater than 35 in all groups were considered as undetected. The PCR data were analysed using web-based PCR array data analysis software (http://pcrdataanalysis.sabiosciences.com/pcr/arrayanalysis.php-). The raw miRNA data was normalized using a global normalization method.

### Target prediction and pathway analysis

Target Scan 6.2 and miRDB were used to predict the target genes of the differentially expressed miRNAs in both follicular fluid and blood plasma of hyperstimulated and unstimulated groups. The predicted miRNA target genes were analyzed by using the DAVID Bioinformatic Resource (http://david.abcc.ncifcrf.gov/) server for Annotation, Visualization, and Integrated Discovery to identify the pathway distribution. These pathways were presented according to the Kyoto Encyclopedia of Genes and Genomes (KEGG) database (http://www.genome.jp/ kegg/). Fisher’s exact test was used to calculate a p-value determining the probability that each biological function or canonical pathway assigned to the data set. In addition, the significance of the association between the data set and the canonical pathway was calculated as the ratio of the number of genes from the data set that were mapped to the pathway divided by the total number of genes that mapped to the canonical pathway.

### Exosome isolation

Exosomes were isolated from 400 μL follicular fluid and blood plasma using differential ultra-centrifugation as previously described [15] with some modification. Briefly, samples were subjected to centrifugation at 4,000 × g for 10 min to remove cell and cell debris. Cell free plasma samples were then filtered through 0.22 μm screen to remove particles larger than 200 nm. Purified plasma were diluted into 4 mL of DPBS (life technologies, paisley, UK) solution and mixed gently. Samples were ultracentrifuged in 5-mL (13 × 51 mm) polyallomer tubes (no. 326819; Beckman Coulter) at 120,000 × g for 70 min at 4 °C in a swinging-bucket SWTi 55 rotor. The resulting supernatant was removed and exosomes pellets were resuspended in DPBS solution and centrifuged again at 120,000 × g for 70 min at 4 °C. Finally, the resulting pellet was resuspended in 200 μL DBPS and stored for further use.

### Immunoprecipitation Ago2 protein complex

A total of 400 μL of plasma was used from each group to immunoprecipitate Ago2 protein in order to isolate Ago2 coupled miRNAs. Each plasma sample was diluted with 400 μL of DPBS (pH 7.4) resulting 800 μL of diluted plasma. Following this, 400 μL of Magna Bind goat anti-mouse IgG Magnetic Bead slurry (Thermo Scientific, Rockford, USA) were washed in DPBS and incubated with 10 μg of mouse monoclonal anti-Ago2 mouse normal IgG (Santa Cruz Biotechnology Inc, USA) antibodies for 2 h at 4 °C. The pre-incubated beads and antibody were then added to the 800 μL of diluted plasma and incubated overnight at 4 °C. Beads were washed three times with 1 % Nonidet P-40 buffer (1 % Nonidet P-40, 50 mM Tris–HCl, pH 7.4, 150 mM NaCl, 2 mM EDTA (BD Bioscience, Heidelberg, Germany). After the final wash 800 μL of QIAzol lyses buffer was added directly to the Ago 2 pellet and processed for RNA isolation.

### Western blot analysis

Exosomal and Ago2 immunoprecipitate proteins were isolated from organic phenol part during total RNA isolation using miRNeasy mini kit (Qiagen, Hilden, Germany) and resuspended in 8 M Urea. Approximately 20 μg of protein from each sample were resolved in 12 % SDS-PAGE polyacrylamide gel (Bio-Rad, Corp., Hercules, CA, USA) and immune reactive proteins were visualized with a Chemidoc XRS (Bio-Rad) instrument. CD63 (ExoAB Antibody, SBI, CA, USA) was used to check the efficiency of exosomes isolation, while Ago2 antibody (Santa Cruz Biotechnology Inc, USA) was employed to detect the efficiency of Ago2 immunoprecipitation.

### Individual qRT-PCR Assays

Based on their enrichment in follicular fluid and blood plasma from hyperstimulated heifers, 11 candidate miRNAs were selected for further study. Moreover, high merit was given to those miRNAs which has sequence similarities between human and bovine during the selection of candidate miRNAs. Primer sets of individual qRT-PCR assays for miRNAs: miR-212, miR-182, let-7 g, miR-100, miR-877, miR-200c, miR-221, miR-103, miR-134, miR-147 and miR-127-3p were obtained from Exiqon (Vedbaek, Denmark). Reverse transcription reaction products were combined with SYBR Green master mix (Exiqon, Vedbaek, Denmark) and loaded into the 96-well plates preloaded with individual miRNA primers. Quantitative PCR was run in a Step one plus real time PCR instrument (Applied Biosystems). Relative expression of each miRNA was analysed using a comparative Ct (2^-ΔΔCT^) method and global normalization strategy was employed to normalize the data.

### Statistical analysis

All experiments were performed a minimum of three times. When two groups were compared (i.e., synchronised vs. super ovulated) a Student’s *t*-test was used to detect differences between treatment groups. A *P value of* ≤ 0.05 was considered to be significant. Data are expressed as mean ± SD of replicates.

## Results

### Progesterone profile of experimental heifers

**Fig. 1:**
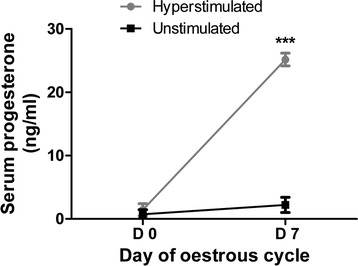
Progesterone profile of experimental heifers: Serum progesterone concentrations (ng/mL) for unstimulated controls and hyperstimulated heifers. Data is presented as mean ± SD and *** designated a significance level of *p* < 0.001

Blood serum progesterone (P4) concentration at day 7 of the experimental heifers was used to identify animals with improper stimulation phenotype. Of the 6 hyperstimulated heifers, while 4 have shown a significantly elevated P4 level (>24 ng/mL) and considered as properly stimulated, 2 heifers (P4 concentration < 23 ng/mL) considered as improperly stimulated and excluded from the experiment. The progesterone concentration in blood serum of the selected hyperstimulated and unstimulated heifers at days 0 & 7 is presented in Fig. 1. The mean progesterone concentration at day 7 was 25.17 ± 1.01 and 2.21 ± 1.19 ng/ml (p < 0.001) in the hyperstimulated and unstimulated heifers, respectively.

### Detection of miRNAs in bovine follicular fluid and blood plasma

**Fig. 2:**
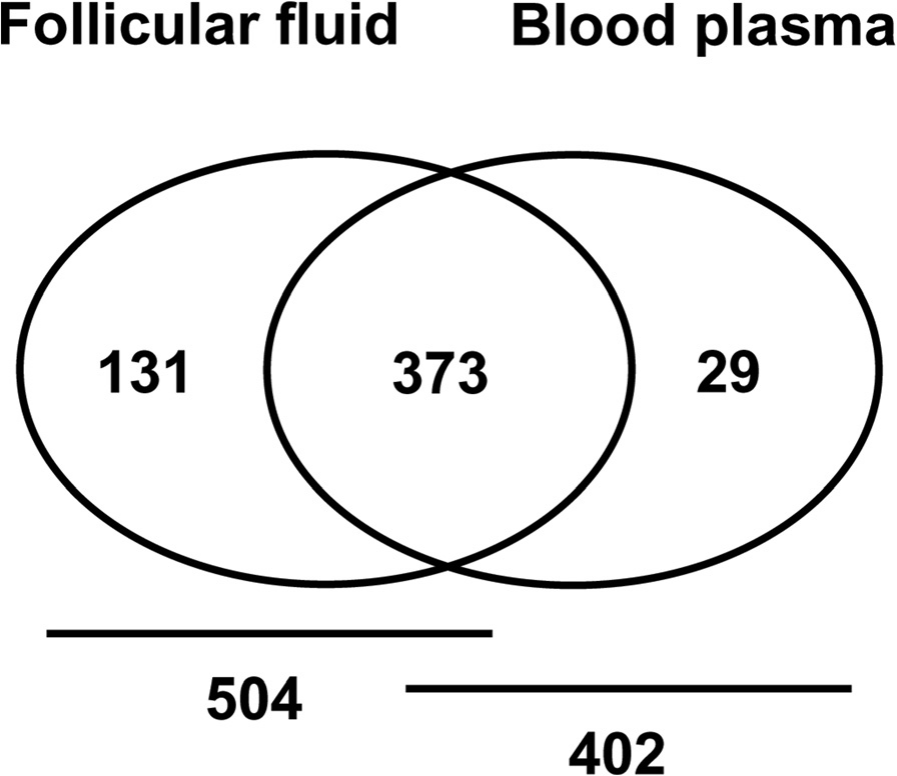
Venn diagram showing the number of detected miRNAs in follicular fluid and blood plasma. Out of 748 miRNAs investigated in the PCR panel I + II, a total of 504 and 402 miRNAs were detected (with threshold cycle value of ≤35 in real time PCR analysis) in follicular fluid and blood plasma, respectively

Here, we examined the abundance of mature miRNAs in follicular fluid and blood plasma of hyperstimulated and unstimulated groups using ready-to-use Human miRNA Panels (I + II) (Exiqon, Denmark) covering 748 mature human miRNAs. After comparative analysis between mature human miRNA sequences used in PCR panels and bovine mature miRNA sequence available in miRBase version 19 (http://www.mirbase.org/), around 241 miRNAs were found to be completely identical, while 126 miRNAs showed differences in size due to addition or deletion of 1–5 nucleotides and 58 miRNA sequences contained mismatches. However, no bovine homologous sequences were matched for 323 miRNAs in the present miRBase databank. A miRNA was considered to be detected when its Ct value is less than 35 and present in at least in 75 % of the samples. Of the 748 miRNA investigated, while 504 miRNAs were detected in follicular fluid, 402 miRNAs were detected in blood plasma (Fig. 2). Among the detected miRNAs 373 miRNAs were commonly detected in both follicular fluid and blood plasma. However, 131 and 29 miRNAs were detected exclusively in follicular fluid and blood plasma, respectively.

### Differential expression of extracellular miRNAs in follicular fluid from hyperstimulated and unstimulated heifers

After excluding 244 miRNAs (31.8 %) with a Ct value above 35 from the dataset, 504 informative miRNAs were analysed in follicular fluid samples. In order to compare miRNA expression differences between follicular fluid from hyperstimulated (*n* = 4) and unstimulated (*n* = 4) animals, first the raw data was normalized using global normalization method (correction with the sum of the expression levels of detected miRNAs), as it was successfully used in previous study compared to spiked-in miRNA or mammalian U6 [31]. Expression analysis of the detected miRNAs in follicular fluid revealed that 57 miRNAs are differentially expressed between two groups (with a fold change of ≥2 & p ≤ 0.05). Of these, 30 miRNAs (52.6 %) were found to be up-regulated, while 27 miRNAs (47.4 %) were down-regulated in hyperstimulated animals compared to the unstimulated ones. Among the up-regulated miRNAs, miR-212 and miR-148b-5p showed the highest fold change regulation. On the other hand, miR-100 and miR-877 showed the greatest fold change regulation among the downregulated miRNAs (Table 1).

**Table 1:** Differential expression of extra miRNAs in follicular fluid and blood plasma obtained from hyperstimulated and unstimulated heifers

Follicular fluid (Hyp vs. Unst)			Blood plasma (Hyp vs. Unst)		
MicroRNA Name	Fold change	*P* value	MicroRNA Name	Fold change	*P* value
miR-643	51.36	0.032678	miR-708-3p	8.15	0.038772
miR-212	40.19	0.007121	miR-26b-3p	4.92	0.031749
miR-148b*	33.13	0.009156	miR-598	3.69	0.002565
miR-548j	23.84	0.031322	miR-423-3p	2.77	0.045389
miR-132	22.45	0.033763	miR-130b-5p	2.73	0.022855
miR-224*	13.98	0.008991	miR-576-5p	2.69	0.024159
miR-129-3p	11.4	0.005949	miR-221	2.66	0.016328
miR-33b	10.57	0.000110	miR-1468	2.63	0.016305
miR-202*	6.53	0.000805	miR-18a-3p	2.55	0.031849
miR-155*	6.51	0.039305	miR-181c	2.40	0.033951
miR-374a	5.2	0.040696	let-7 g	2.00	0.008124
miR-1207-5p	5.11	0.000203	miR-103	2.29	0.002404
miR-182	4.81	0.047938	miR-34a	2.13	0.023235
miR-103	4.49	0.006078	miR-125b	−3.07	0.013367
let-7 g	4.23	0.012259	miR-410	−3.2	0.038659
miR-106b	3.96	0.034579	miR-127-3p	−3.22	0.000817
miR-191	3.95	0.021379	miR-494	−3.23	0.008614
miR-542-5p	3.78	0.005687	miR-147	−3.63	0.047214
miR-505*	3.57	0.006276	miR-99a*	−5.54	0.044064
miR-550a	3.19	0.046925	miR-134	−7.03	0.043678
miR-107	3.03	0.034726	miR-153	−7.21	0.006251
miR-595	2.78	0.030403			
miR-33a*	2.74	0.042360			
miR-374b	2.65	0.038757			
miR-30b	2.62	0.039581			
miR-31	2.54	0.029654			
miR-106b*	2.43	0.005030			
miR-940	2.37	0.014177			
miR-495	2.27	0.010936			
miR-494	2.06	0.046091			
miR-300	−2.08	0.020863			
miR-24	−2.09	0.000273			
miR-452	−2.15	0.020850			
miR-151-5p	−2.22	0.033121			
miR-320a	−2.58	0.027659			
miR-125a-3p	−2.78	0.008997			
miR-99a	−2.94	0.048804			
miR-449a	−2.97	0.018677			
miR-206	−3.19	0.010009			
miR-23a	−3.26	0.007032			
miR-27b	−3.44	0.013330			
miR-139-5p	−3.46	0.006195			
miR-125b-2*	−3.6	0.029345			
miR-378	−3.72	0.017333			
miR-125b	−3.86	0.017793			
miR-141	−4.03	0.038193			
miR-23b	−4.11	0.033330			
miR-190	−4.2	0.004785			
miR-382	−4.45	0.035549			
miR-92a	−4.53	0.007967			
miR-145	−4.78	0.000015			
miR-361-5p	−5.25	0.029699			
miR-200a*	−6.41	0.040837			
miR-200c	−6.48	0.048195			
miR-659	−8.42	0.037679			
miR-877	−12.16	0.009853			
miR-100	−14.02	0.004324			

### Expression profile of circulatory miRNAs in blood plasma derived from hyperstimulated vs. unstimulated heifers

The same PCR array platform was used to compare the pattern of circulatory miRNA expression in blood plasma at day 7 of oestrous cycle between two experimental groups. By employing the same criteria for data filtration, a total of 402 (54.8 %) informative miRNAs were identified in blood plasma samples of both groups. Among the detected miRNAs, 21 miRNAs were identified as differentially expressed (p < 0.05), of which 13 were overexpressed and 8 were underexpressed in the plasma of hyperstimulated heifers (Table 1). The miRNA expression in the hyperstimulated plasma compared to the unstimulated control was increased by 2 to11-fold but decreased by 2.8to 7-fold (Table 1). Among the overexpressed miRNAs miR-20b-3p and miR-708-3p showed the highest fold change regulation, while miR-153 and miR-134 exhibit highest fold change among the downregulated miRNAs.

### Cluster of miRNAs affected by ovarian hyperstimulation in follicular fluid and blood plasma

**Fig. 3:**
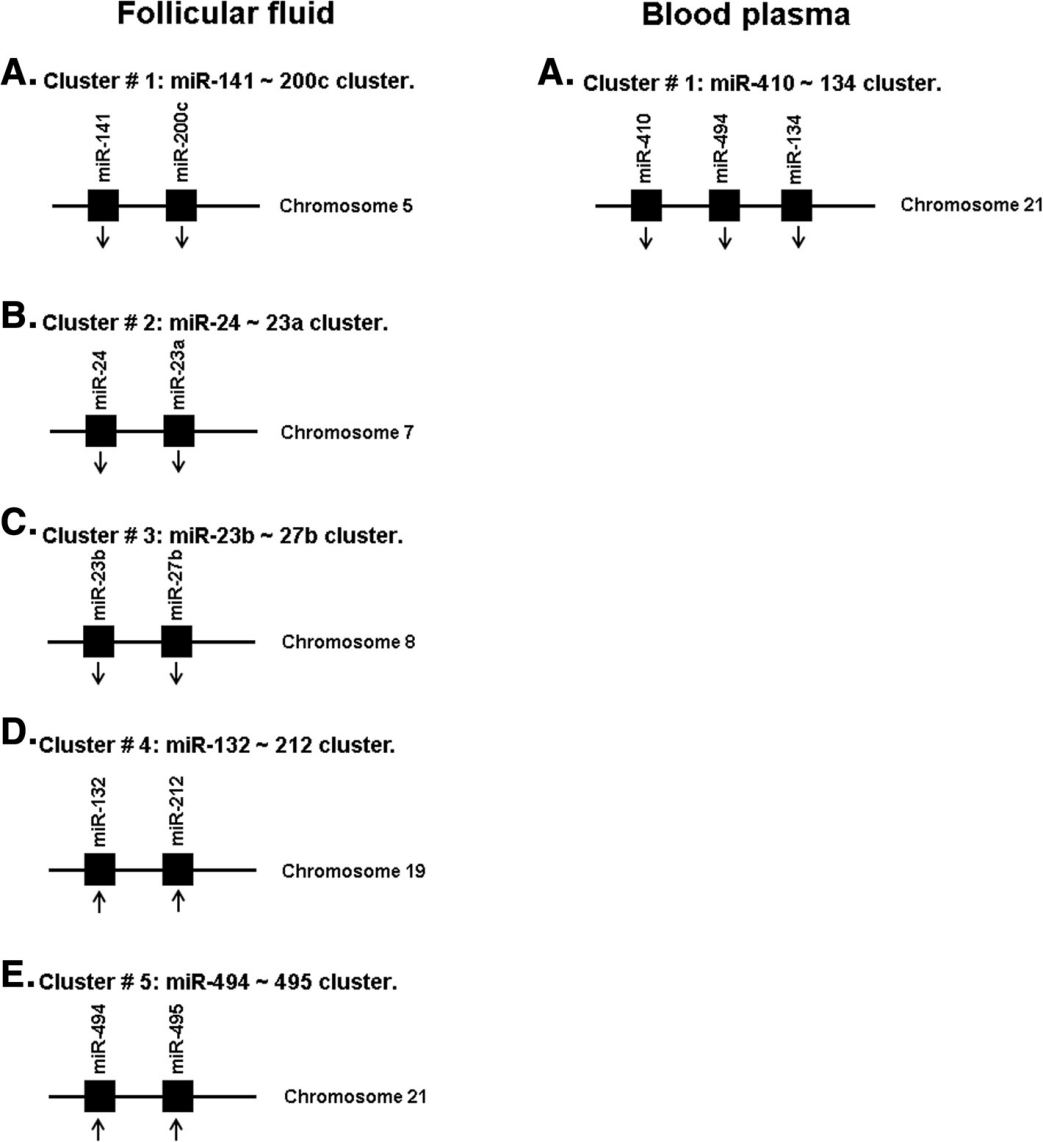
The number of miRNAs in each cluster and their distribution in bovine chromosomes. While clusters differentially expressed in follicular fluid (**a**-**e**) comprise two miRNAs, clusters in blood plasma shown to comprise three miRNAs. Multiple miRNA clusters are affected in the follicular fluid and blood plasma due to ovarian hyperstimulation. The number of miRNA genes in each cluster ranged from 2-3. In all clusters, the expression of miRNA genes in a particular cluster was in the same direction (the arrow **↑** for increased expression, and the arrow **↓** for decreased expression). Please note that differentially expressed miRNAs (≥2 fold and *p* < 0.05) in follicular fluid and blood plasma of hyperstimulated heifers compared to the un stimulated ones are listed in Table 1

MiRNA cluster represent group of miRNA genes located within 10 Kb of distance on the same chromosome. To test the hypothesis that controlled ovarian hyperstimulation may affect the expression of individual or cluster miRNAs, the genomic location of the 57 and 21 miRNAs that were differentially expressed in follicular fluid and blood plasma of hyperstimulated heifers, respectively, were determined. Subsequently, we found that approximately 18 % of the follicular fluid derived differentially expressed miRNAs to belong to miRNA clusters and the rest 82 % were individual miRNAs (Fig. 3). Of those in blood plasma differentially expressed miRNAs, 15 % were found to be present in clusters. Variable numbersof miRNAs were present in each cluster with minimum of two. For example, the miRNA cluster miR-410 ~ 134, which is found to be differentially expressed between hyperstimulated and unstimulated heifers blood plasma, contained 3 miRNAs. Since miRNAs within the cluster may share a common gene promoter and may be regulated as a single transcriptional unit, it could be of interest to determine whether any entire cluster changed in the same direction following treatment. Subsequently, we found that all miRNAs within all 6 clusters (Fig. 3) showed the same direction of expression tended to change in the same direction as the other members in that cluster.

### miRNA target prediction and pathway analysis

As the number of experimentally validated targets of miRNA is limited, we used the widely used TargetScan and miRDB algorithm to obtain the list of genes predicted to be targeted by the differentially expressed miRNAs obtained from follicular fluid and blood plasma of the experimental animals. Accordingly, 8311 and 8050 genes were predicted to be targeted by up- and down-regulated miRNAs, respectively, between follicular fluid from hyperstimulated vs. unstimulated heifers. To overcome the limitation of DAVID Bioinformatic Resource (limited processing ability: 3,000 genes), only top 100 genes were considered for each miRNA for further bioinformatics analysis. Accordingly, after screening of the long list of genes, 3000 and 2700 genes were identified as potential targets of up- and down-regulated miRNAs, respectively, in follicular fluid samples. Similarly, a total of 6267 and 4982 genes were found to be potentially targeted by up- and down-regulated miRNAs in blood plasma samples of the experimental animals. Following screening, a total of 1300 and 800 genes were identified as potential targets of up- and down-regulated miRNAs, respectively, in blood plasma samples of hyperstimulated versus unstimulated heifers. Finally the screened list of genes from both follicular fluid and blood plasma samples were subjected to a pathway analysis using NCBI DAVID Bioinformatic Resource 6.7 to identify the significantly enriched canonical pathway (*P* < 0.05).

As shown in Table 2 and Table 3, among the several list of pathways, MAPK signalling pathway and Wnt signaling pathway are the most enriched pathways potentially regulated by the differentially expressed miRNAs. In addition, pathways involved in various signal transductions and cell-to-cell interaction including *ErbB signaling pathway*, *Axon guidance, Neurotrophin signaling pathway, Endocytosis, Oocyte meiosis, TGF-beta signaling pathway* and *Focal adhesion* were among the most enriched pathways for genes potentially targeted by miRNAs derived from both follicular fluid and blood plasma of hyperstimulated heifers. Taken together, these bioinformatics exploratory analyses suggest that variation in the level of circulatory miRNAs in follicular fluid and blood plasma might affect critical pathways involved in follicular development and oocyte maturation. As miRNAs target prediction algorithms are known to contain both false positives and false negatives, and our in silico pathway enrichment analysis is based on mRNA genes predicted to be targeted by extracellular miRNAs, a full understanding of the potential functional role of extracellular miRNAs can only be established using functional experiments.

**Table 2:** List of enriched pathways (*p* <0.05), in which genes predicted to be targeted by differentially expressed miRNAs (*P* <0.05) between follicular fluid from hyperstimulated vs. unstimulated heifers

Pathway	miRNAs involved	*P* value	Top genes
Overexpressed miRNAs (hyp vs. unst)			
TGF-beta signaling pathway	miR-106b, −132, −148b-5p, −182, −212, −374a, −548j	1.75E-07	TGFBR2, TGFB2, SMAD2, SMAD3, SMAD4, BMPR2
Axon guidance	miR-22-5p, −30b, −31, −33a-3p, −182, −132, −550a	2.85E-06	EFNB1, DCC, EPHB4, EPHA3, PLXNA1, PAK4
MAPK signaling pathway	miR-30b, −106b, −132, −182, −212, −548j, −202-5p	6.43E-05	MAP3K1, MAP3K5, KRAS, MRAS, GRB2, FGF7
Endocytosis	miR-33a-3p, −106b, −182, −374a, −374b, −202-5p	3.83E-04	RAB11FIP4, RAB11FIP2, EEA1, IGF1R, EPS15, EPN
Colorectal cancer	miR-30b, −33a-3p, −106b, −132, −212, −384, −494	5.94E-04	SOS1, FZD3, SMAD2, DCC, MAPK1, BAX
Pathways in cancer	miR-33a-3p, −107, −132, −212, −494, −495, −548j	5.95E-04	E2F1, FGF18, WNT16, FGF7, PTEN, MITF
Wnt signaling pathway	miR-132, −212, −33a-3p, −494, −940, −495, −548j, −107	6.73E-04	LRP5, LRP6, TCF7, PLCB4, DVL3, WNT1, WNT5A
Neurotrophin signaling pathway	miR-106b, −30b, −940, −182, −212, −107,	0.001267	PIK3R2, NTRK2, NTRK3, RPS6KA6, IRS1, RAC1
Oocyte meiosis	miR-212, −132, −940, 495, −595, −107	0.003369	CDC27, CPEB1, PRKACB, FBXW11, MAPK1, RPS6KA3
GnRH signaling pathway	miR-940, −495	0.007085	PRKCA, SRC, MAP3K2, MAPK14, GRB2, MAP3K3
Underexpressed miRNAs (Hyp vs. Unst)			
Pathways in cancer	miR-659, −141, −190, −449a, −200c, −361-5p, −145	9.51E-12	KRAS, PTEN, VEGFA, STAT5B, MITF, BCL2
MAPK signaling pathway	miR-23a, −23b, −141, −145, −200c, −92a, −125b, −24	2.94E-11	MKNK2, MEF2C, FGF, SOS1, RAS, MAerkPK2
Wnt signaling pathway	miR-125b, −449a, −302e, −145, −23a, −29b-2-5p, −659, −200c	2.76E-08	LRP5, LRP6, TCF7, PLCB4, DVL3, WNT1, WNT5A
ErbB signaling pathway	miR-23a, −23b, −125b, −206, −302e, −513c	3.19E-07	ERBB4, GRB2, SHC1, PIK3R3, PIK3CD, AKT3
Colorectal cancer	miR-659, 23a, −23b, −141, −190, −449a, −200c, −361-5p, −145	4.48E-07	DCC, APPL1, TGFBR2, SMAD3, SMAD4, APC
Axon guidance	miR-23a, −23b, −200c, −145	7.23E-07	PLXNC1, EFNB2, PAK4, DCC, SEMA6A, PAK7, MET
Neurotrophin signaling pathway	miR-302e, −361-5p, −452, −525-5p, −449a, −659	1.82E-06	NTRK2, NGFR, KRAS, MAP3K1, PIK3R3, PLCG1
Renal cell carcinoma	miR-659, −141, −190, −449a, −200c, −361-5p, −145	3.75E-05	GAB1, MET, SOS1, GRB2, VEGFA, NRAS
Focal adhesion	miR-24, −27b, −125a-3p, −139-5p, −206, −200c	5.04E-05	ITGB3, PXN, SHC3, ACTB, SRC, PAK2
Regulation of actin cytoskeleton	miR-200c, −145, −27b, −92a, −300, −206	8.29E-05	RAC1, ROCK2, GIT1, PIK3R3, ITGA3, LIMK1

**Table 3:** List of enriched pathways (*P* < 0.05), in which genes predicted to be targeted by differentially expressed miRNAs (*P* < 0.05) in blood plasma of hyperstimulated vs. unstimulated heifers

Pathway name	miRNA involved	*P* value	Top genes
Overexpressed miRNAs (hyp vs. unst)			
Pathways in cancer	let-7 g, miR- 20b-3p, −22, −26b-3p, −34a, −221, −181c	1.44E-09	AKT3, PIK3R3, RASSF1, KRAS, MEK, DCC
Wnt signaling pathway	let-7 g, miR-221, −32	4.04E-08	WNT1, SMAD2, DKK2, DVL3, VANGL2, CCND1
Neurotrophin signaling pathway	let-7 g, miR-22, −181c, −221	6.73E-08	NTRK2, IRS2, AKT3, NGF, PIK3CD, NRAS
Axon guidance	miR-34a, −181c, −221	2.19E-07	PLXNC1, DPYSL2, PAK1, MET, KRAS, EFNB1
Endocytosis	miR-22, −34a, −130b-3p, −576-5p	1.23E-06	TGFBR1, RAB5A, RAB11FIP4, PDCD6IP, VPS37A
MAPK signaling pathway	let-7 g, miR-22, −32, −34a, −221	2.69E-06	RRAS, MAP3K1, MAPK1, MAPK9, SRF, MAP2K4
Colorectal cancer	let-7 g, miR-22, −26b-3p, −24-2-5p	4.59E-06	DCC, TGFBR1, KRAS, MAP2K1, AKT3, CASP3
Chronic myeloid leukemia	miR-22, −181c, −221	4.98E-06	NRAS, AKT2, BCR, MAPK1, GAB2, BCL2L1
TGF-beta signaling pathway	miR-26b-3p, −221, −22, −32	9.63E-06	ACVR2A, TGFBR2, SMAD2, SMAD4, BMP7
ErbB signaling pathway	miR-22, −181c, −221	2.96E-05	ERBB4, GAB1, MAP2K1, PAK4, PIK3CD
Underexpressed miRNAs (Hyp vs. Unst)			
Pathways in cancer	miR-125b, −153, −410, −494	9.51E-12	DCC, GRB2, SMAD2, SOS1, E2F3
MAPK signaling pathway	mir-125b, −153, −494	2.94E-11	MEF2C, MAPKAPK2, MAP3K1, TGFB2, FGFR2
Wnt signaling pathway	miR-147, −153, −410, −494	2.76E-08	LRP6, WNT5A, DVL3, DKK2, PLCB1, ANGL2
ErbB signaling pathway	miR-125b, −410	3.19E-07	ERBB4, SOS1, MAPK1, NRG3, GAB1, MAP2K7
Colorectal cancer	miR-125b, −410, −153, −494	4.48E-07	DCC, BCL2, SMAD4, RAF1, SMAD2, PIK3R3
Axon guidance	miR-147, −153, −410, −494	7.23E-07	PLXNA2, ROCK2, EFNA3, NFAT5, MAPK1
Neurotrophin signaling pathway	miR-125b, −134, −147, −494	1.82E-06	NTF3, MAP3K1, SOS1, SORT1, BCL2
Melanogenesis	miR-134, −99a-3p	4.52E-06	MAPK1, WNT5A, KRAS, GNAI3, CREB1
Melanoma	miR-125b, −494	1.70E-05	E2F2, FGFR1, IGF1R, FGF7, RAF1
Prostate cancer	miR-125b, −147, −153, −410, −494	1.82E-05	FGFR2,, IGF1R, MAPK1, IGF1, BCL2

### Temporal expression of candidate circulatory miRNAs in blood plasma across oestrus cycle

**Fig. 4:**
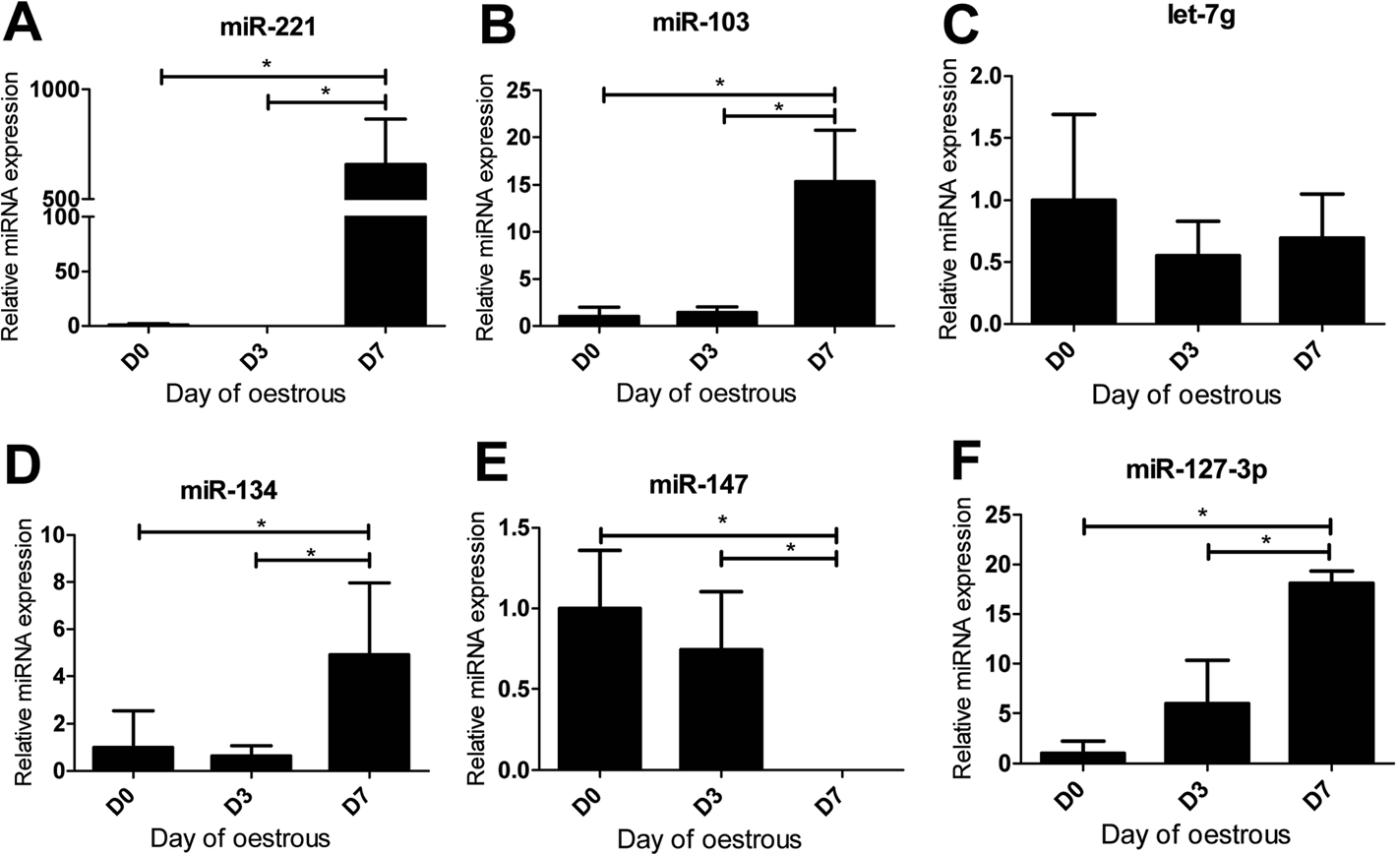
Temporal expression of candidate circulatory miRNAs in blood plasma across estrous days. Relative expression of candidate circulatory miRNAs in blood plasma of hypersitimulated heifers across different days during estrous (**a**-**f**) Expression of miR-221, miR-103, let-7g, miR-134, miR-147 and miR-127-3p was determined by RT-qPCR and each miRNA expression level was normalized using global normalization method. The error bars showed the SD. The significance of differences for miRNA expression was calculated using two-tailed *T*-test and *, *p* < 0.05. D0: onset of estrous; D3: day 3 of estrous; D7: day 7 of estrous

In order to explore the temporal changes in the expression of circulatory miRNA especially in blood plasma of hyperstimulated heifers, the expression of candidate miRNAs was investigated at different days during the estrous cycle (Days 0, 3 and 7). As shown in Fig. 4 expression analysis of candidate miRNAs (miR-221, miR-103, let-7 g, miR-134, miR-147 and miR-127-3p) using qRT-PCR revealed the presence of temporal changes in the pattern of expression depending on the time during the estrous cycle as shown in Fig. 4. While four of the candidates namely. miR-221, miR-103, miR-134 and miR-127-3p showed a significant increase in abundance at day 7 of the estrous cycle compared to days 0 and 3, miR-147 showed a decreasing pattern from day 0 to day 7. No significant difference in the expression of let-7 g miRNA has been observed between the days during estrous cycle.

### Validation of exosomes and Ago2 protein complexes isolated from follicular fluid and blood plasma

**Fig. 5:**
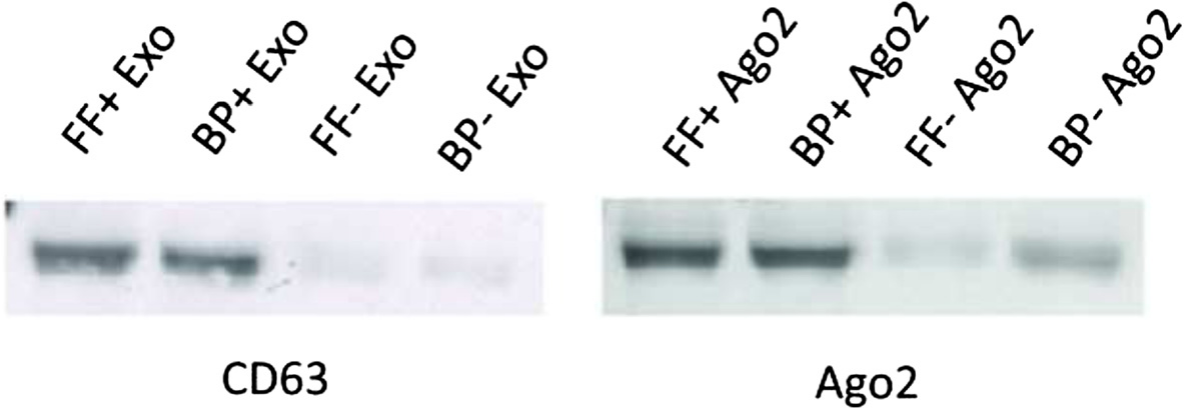
Specificity of isolation of exosomes and Ago2 protein complex from follicular fluid and blood plasma. Exosome and Ago2 proteins were isolated from organic-phenol fraction during total RNA isolation using miRNeasy kit and resolved in 8M urea. Protein concentrations were quantified using Bradford assay and total of twenty microgram protein from each group were separated in 12 % SDS-PAGE, transferred nitrocellulose membrane and incubated with specific antibody (CD63 and Ago2). Followed by HRP-conjugated secondary antibody and detected using chemiluminescent substrate. Western-blot results indicate the specificity of exosome and Ago2 protein isolation from follicular fluid (FF) and blood plasma (BP) as indicated in the corresponding figures

To determine the mode of circulation of candidate miRNAs in follicular fluid and blood plasma, the exosomal and Ago2 protein fractions of the biological fluid samples were isolated using differentially ultracentrifugation and immunoprecipitation procedures respectively. The specificity of both isolation procedures was validated using a western blot analysis of exosomes and Ago2 specific marker proteins. Immunoblotting of CD63 protein showed the presence of strong band in the exosome fraction of both follicular fluid and blood plasma samples, while the Ago2 protein band was evident in immunoprecipitated portion of the samples (Fig. 5). Despite ignorable detection of Ago2 protein in exosome fractions, strong bands for CD63 and Ago2 proteins in exosomes and Ago2 fraction, respectively suggest the efficient recovery of exosomes and Ago2 protein complexes from follicular fluid and blood plasma.

### Detection of candidate circulatory miRNAs in exosomes and Ago2 fraction of follicular fluid

**Fig. 6:**
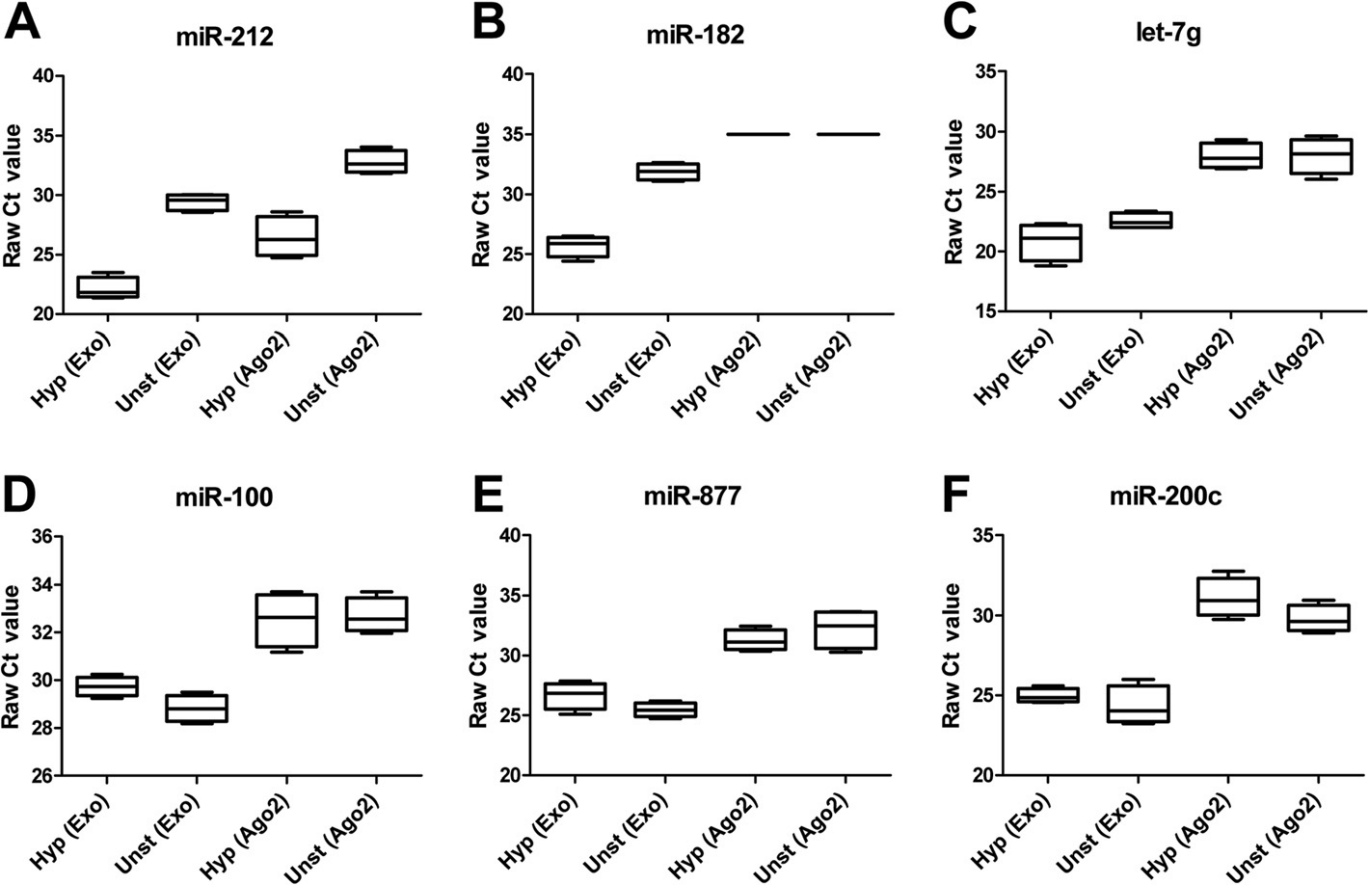
Detection of candidate extracellular miRNAs in exosomes and Ago2 fraction of follicular fluid derived from hyperstimulated and unstimulated heifers. The boxplots on panels a through f represent the detection of candidate extracellular miRNAs in exosomes (Exo) or Ago2 protein complex (Ago2) fraction of follicular fluid samples derived from hyperstimulated (Hyp) and unstimulated (Unst) heifers. The error bars show the SD. Data are presented as raw Ct value and Ct value of more than 35 was considered as undetected

Here we investigate the expression pattern of selected candidate miRNAs in both exosomes and Ago2 fractions of follicular fluid derived from hyperstimulated versus unstimulated heifer groups. For this a total of six miRNAs representing those whose expression was induced (miR-212, miR-182 & let-7 g) or suppressed (miR-100, miR-877 and miR-200c) due to hyperstimulation were selected for detecting their expression in exosome and Ago2 fractions. The level of detection of candidate miRNAs was determined by real time PCR and data are shown as raw Ct value in Fig. 6. As shown in this Fig. 6, except miR-182, all candidate miRNAs were detected in both exosomal and Ago2 fraction of follicular fluid of both hyperstimulated and unstimulated animals. Mir-182 was not detected in Ago2 fraction (with raw Ct value more than 35) in both hyperstimulated and unstimulated heifers.

### Detection of candidate circulatory miRNAs in exosomes and Ago2 fraction of blood plasma

**Fig. 7:**
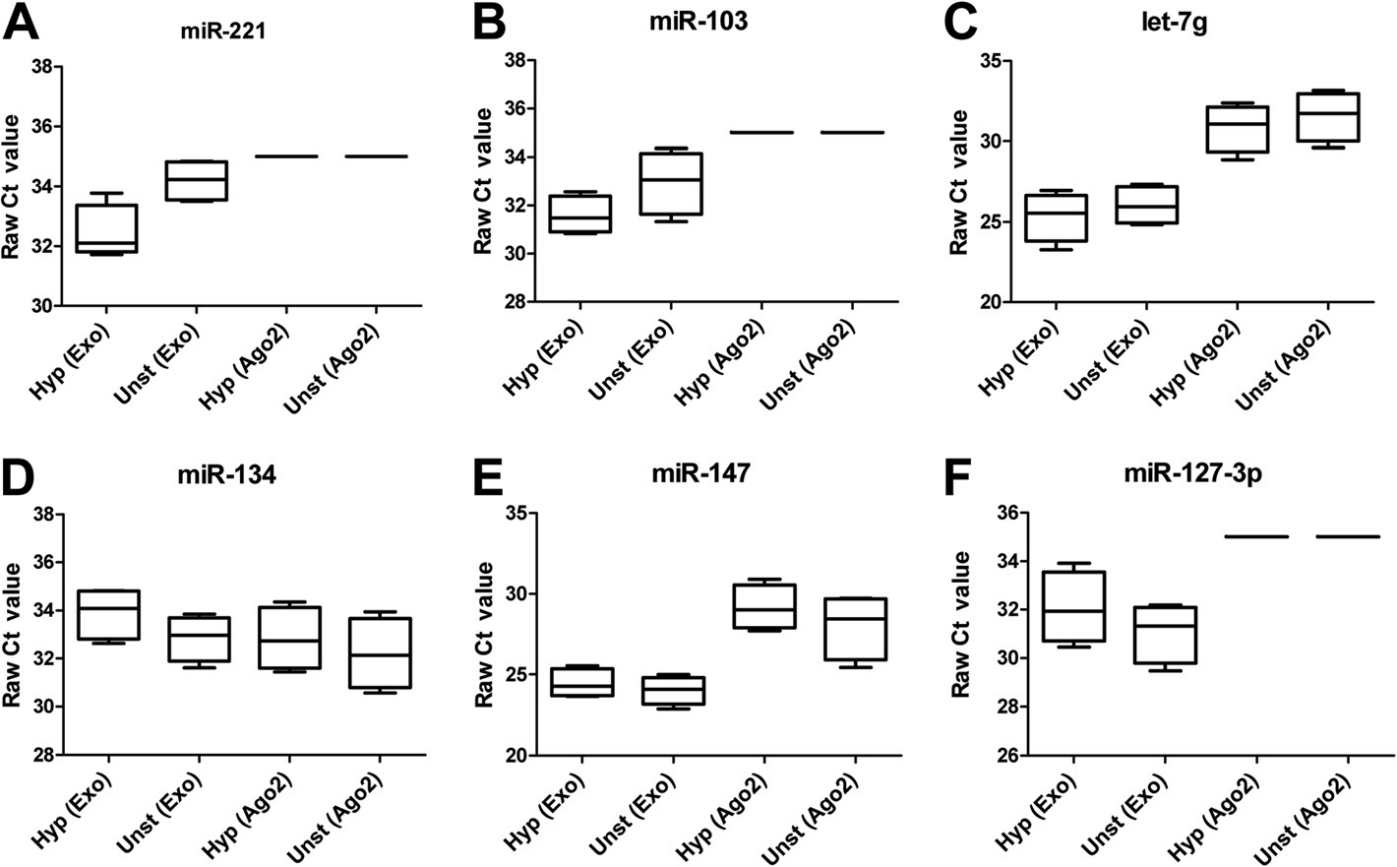
Detection of candidate circulating miRNAs in exosomes and Ago2 fraction of blood plasma derived from hyperstimulated and unstimulated heifers. The boxplots on panels a through f represent the detection of candidate extracellular miRNAs in exosomes (Exo) or Ago2 protein complex (Ago2) fraction of blood plasma samples from hyperstimulated (Hyp) and unstimulated (Unst) heifers. The error bars show the SD of 4 replicates. Data are presented as raw Ct value and Ct value of more than 35 was considered as undetected

Here we have tried to detect candidate miRNAs, which were enriched (miR-221, miR-103 & let-7 g) or suppressed (miR-134, miR-147 & miR-127-3p) in blood plasma samples of hyperstimulated animals compared to the unstimulated ones. As shown in Fig. 7, all candidate miRNAs, except miR-103 and miR-127-3p, were detected in both exosomal and Ago2 fraction of blood plasma of both hyperstimulated and unstimulated heifers. Mir-103 and miR-127-3p were not detected in the Ago2 protein fraction of both treatment groups.

## Discussion

Controlled ovarian hyperstimulation (COH) facilitate growth of multiple follicles in monoovulatory animals. COH involves stimulation of the ovaries by supraphysiological levels of gonadotropins and also associated with very low level of luteinizing hormone during the luteal phase, the peri-implantation and implantation period (Kolibianakis *et al.*, 2006). Thus, the goal of COH is multifollicular recruitment with retrieval of multiple oocytes in an effort to compensate for the “inefficiencies” of the outcome of IVF laboratory. However, several reports showed that, COH itself has adverse effects on oocyte development, embryo quality, endometrial receptivity and perhaps also perinatal outcomes [6, 7, 32] The effect of COH on oviductal environment and subsequently on the transcriptome of the resulting embryos has been evidenced [8].

Recently miRNAs have been detected in extracellular environment mainly in different bio-fluids of various species [15, 25, 33, 34]. Given the emerging association of circulating miRNA species with a spectrum of patho-physiological conditions, we have systematically explored the changes in circulatory miRNAs in follicular fluid and blood plasma induced by controlled ovarian hyperstimulation. The results of this study clearly uphold our primary hypothesis that introduction of supra-physiological level of gonadotropins during the process of COH may induce the alteration of expression of extracellular miRNAs in follicular fluid as well as in blood plasma.

Since their discovery [13, 14], extracellular miRNAs got a research priority as they have been found to be differentially expressed in altered physiological conditions [13]. Recently, it has been shown that a large number of miRNAs are present in bovine follicular fluid and their expression patterns differs depending on the growth status of oocyte [15]. In the present study we profiled extracellular miRNAs in bovine follicular fluid and blood plasma derived from hyperstimulated and unstimulated heifers using Human miRNome PCR array platform. A specific miRNA transcript was considered as detected when its Ct value <35 and present in at least 75 % of the replicates. This selection criteria was set based on the previous experiences in our lab with a similar study design [15]. Similar studies on detection of miRNAs in plasma samples also use the same threshold level to determine the detection level of transcripts [35], even as high as ≤37 cycle as a threshold to determine the level of detection of miRNAs in equine follicular fluid [36]. Despite using a heterologous approach, the quantitative real time PCR analysis revealed a wide array of miRNAs to be present in both follicular fluid and blood plasma, indicating the cross species conservation feature of miRNAs between human and bovine as it has been observed between wide range of species [37]. Of 748 miRNA investigated in the PCR array panel, a total of 504 and 402 miRNAs were detected in follicular fluid and blood plasma, respectively. Comparable detection rate was also reported for follicular fluid [15] and blood plasma [38]. Among the detected miRNAs, 373 were commonly found in both follicular fluid and blood plasma. Results from this study and our previous findings [15] confirmed that, relatively higher number of extracellular miRNAs are present in follicular fluid than blood plasma, which may be attributed to the condensed nature of the former.

By comparing hyperstimulated vs. unstimulated heifers, 57 and 21 miRNAs were found to be differentially regulated in follicular fluid and blood plasma, respectively. Out of 57 differentially expressed miRNAs, 30 miRNAs were found to be up-regulated in follicular fluid of hyperstimulated heifers. Several of these up-regulated miRNAs have been reported to play an important role in follicular development and other physiological processes. For instance, miR-212 & miR-132 were reported to be involved in regulating follicular development [39, 40]. Moreover, these two miRNAs (miR-212, −132) were found to be significantly up-regulated in preovulatory mouse mural granulosa cells following LH/hCG induction [41] indicating their potential involvement in follicular development in those species. The miR-148*, a minor form of miR-148, was also significantly up-regulated in follicular fluid of hyperstimulated heifers. Although, the role of this miRNA in follicular development is unclear, miR-148/152 family was found to be important for growth and development of normal tissue [42]. Interestingly, miRNAs like miR-643, miR-548j, miR-224* and miR-33b, which were found to be highly abundant in follicular fluid of hyperstimulated heifers, are also reported to be highly associated with cancer [43–46]. Among the down-regulated miRNAs, miR-361-5p [47] and miR-27b (homologous to miR-27a, sharing 20 out of 21 nts) [48] were reported as tumor suppressors. Despite accumulated evidences on the hormonal regulatory role of cellular miRNAs and vice versa [41], the present study is the first of its kind reporting the effect of suprarphysiological level of gonadotropins on the level of circulating miRNAs in bovine follicular fluid and blood plasma. However, the consequence of this alteration on the resulting oocytes, embryos and further ovarian function needs further investigation.

In blood plasma, 21 miRNAs were found to be differentially expressed between hyperstimulated and unstimulated heifers where 13 miRNAs were up-regulated and 8 miRNAs were down regulated. Among the up-regulated miRNAs miR-708-3p, which showed the highest fold change regulation, was reported in promoting the development of bladder carcinoma via direct repression of Caspase-2 [49]. Several studies have shown the association of miR-26b, which was induced in expression due to hyperstimulation, with pregnancy-associated disorder like preeclampsia [50]. The potential association of this miRNA in reproductive disorder due to hyperstimulated ovaries cannot be ruled out. Interestingly, miR-26b was also reported to be elevated in plasma from severe preeclamptic pregnancies [51]. MiR-221, which is commonly known as oncomir, was also significantly elevated in blood plasma of hyperstimulated heifers. In the present study we also found a panel of down regulated miRNAs including miR-153 and miR-134 which are commonly known as tumor suppressor miRNA [52, 53].

Among the differentially expressed miRNAs in follicular fluid and blood plasma of hyperstimulated heifers, 13 miRNAs belong to 6 miRNA clusters suggesting the importance of understanding the structure and function of these miRNAs as clusters. miRNA clusters, which are groups of tandem miRNA genes that are closely located in the genome, are abundantly and widely distributed in animal genomes. It has been revealed that about 50 % of the miRNA genes in *Drosophila* [54] and over 30 % of the miRNA genes in human, mouse, rat and chicken are located in the genome as clusters [55]. The length of the transcript typically varies from a couple of kb to over 10 kb. It has been reported that each miRNA cluster usually has up to 10 miRNA genes [56]. Many of the miRNA clusters may have one core promoter region and transcriptional start site shared by all miRNA genes within that cluster and are ultimately expressed within a single RNA transcript [57]. Co-expression experiments of clustered miRNA genes showed that one miRNA cluster is usually transcribed as a single transcriptional unit [58] as a result of some kind of regulatory coordination within the clustered miRNAs. In the present study, we found that the miR-134 ~ 410 cluster which comprise 3 miR genes, while rest of the clusters has 2 miR genes per cluster. Although several miRNA clusters have been reported to be associated with pathological and physiological conditions in human and mice, the overall expression pattern of extracellular miRNA clusters during follicular development has not been extensively studied. We have recently identified several classes of cluster miRNAs in bovine follicular granulosa cells derived from subordinate and dominant follicles during folliculogenesis [59, 60]. Since many miRNA genes within a cluster share a common promoter or utilize common regulatory machinery, examination of their expression pattern and regulatory mechanism could enhance our understanding of the role of extracellular miRNAs in the process of follicular development.

Another interesting observation from our study is that the number of differentially expressed miRNAs in follicular fluid is higher (57) than blood plasma (21) of hyperstimulated heifers. This is may be due to the fact that gonadotropins action is specific to the target organs [61]. It has been well accepted that miRNAs are involved in hormone regulation or vice versa [41]. FSH controls the development of granulosa cells during folliculogenesis by stimulating their proliferation and differentiation, and by promoting the formation of the follicular antrum. In particular, FSH enhances the biosynthesis of female sex hormones estradiol and progesterone, which control the estrous cycle and reproduction [62, 63]. Numerous studies have shown that the LH surge initiates the transcriptional upregulation and downregulation of genes, including cytokines, transcription factors, and matrix-remodeling proteins, within periovulatory granulosa cells [64, 65]. Therefore, the higher number of differentially regulated miRNAs in follicular fluid of hyperstimulated heifers may indicate the higher transcriptional activity of follicular cells within follicular microenvironment during the process of COH.

In addition to their expression level, we have investigated the biological relevance of these differentially regulated miRNAs. For this the target genes of differentially expressed miRNAs were predicted bioinformatically and the dominant biological pathways were determined. The most significant pathways enriched by predicted targets for up-regulated miRNAs in follicular fluid of hyperstimulated heifers including TGF-beta signaling pathway, Axon guidance, neurotrophin signaling, MAPK signaling colorectal cancer and pathways in cancer. All these pathways are known to be involved in ovarian follicular growth, different developmental processes and also in several pathological conditions. MiRNAs differentially expressed in plasma samples were found to be involved several pathways including cancer, WNT signaling pathway Axon guidance, MAPK signaling pathways colorectal cancer etc. Although the miRNA expression profiles were different from each other, *in silico* predicted biological pathways were similar between follicular fluid and blood plasma. This is consistent with the observation that the course of ovarian hyperstimulation may be characterized by certain common pathways in the whole physiology of the animal. However, it is important to note that our observations for pathways are exploratory in nature and need further validation using wet-lab studies to elucidate miRNA-mRNA interaction and causal association of differentially expressed miRNAs and the predicted pathways.

So far, it has been established that miRNAs are expressed spatio-temporally in different developmental process [66] and also in adult tissues [67]. A study conducted with sheep ovarian tissues demonstrated differential expression of miRNA in follicles and corpora lutea during the ovine oestrous cycle [68]. This study demonstrated that eight miRNAs (miR-503, miR-21, miR-29b, miR-142-3p, miR-34a, miR-152, miR-25 and miR-130a) were highly expressed, while nine miRNAs (miR-125a, miR-199a-3p, miR-125b, miR-99a, let-7c, miR-145, miR-31, miR-202 and miR-27b) were expressed at lower level between the follicular and luteal stages in ovine ovarian tissues. In the present study temporal expression of miRNAs was observed in blood plasma of hyperstimulated heifers across estrous cycle. For instance, miR-221, miR-103, miR-134 and miR-127-3p show significantly higher expression at day 7 of estrous cycle compared to day 0 or day 3. In contrast, miR-147 has significantly lower expression at day 7 of estrous. However, there is no significant difference in the expression of let-7 g across the estrous days.

Several studies have demonstrated that circulating miRNAs may be coupled either with exosomes [21, 25] or Ago2 protein complex [28] to bypass the high RNases activity in blood stream or in follicular fluid. To proof the mode of circulation of those miRNAs in follicular fluid and blood plasma we have detected the expression of candidate miRNAs in exosomal and Ago2 protein fractions of both follicular fluid and blood plasma of heifers in both treatment groups. The specificity of isolation of exosome and Ago2 fraction was confirmed by western blot analysis of CD63 and Ago2 protein [15, 69]. Candidates including miR-182 from follicular fluid (Fig. 4) and miR-221, miR-103 and miR-127-3p from blood plasma (Fig. 5) were found to be detected only in exosomal fraction and completely undetected in the Ago2 fraction. In both follicular fluid and blood plasma samples, all miRNA which were detected in both fractions, showed higher abundance in exosomal fraction compared to the Ago2 protein complex. This is in agreement with our previous findings showing that exosome mediated release of miRNAs to be the dominant pathway in follicular fluid [15]. Interestingly, the hyperstimulation treatment had no effect on the detection of candidate miRNAs either in exosomal or Ago2 protein complexes.

## Conclusion

The present study demonstrated the consequence of ovarian hyperstimulation in inducing changes in the relative abundance of extracellular miRNAs, which are potentially involved in regulation of genes involved in several physiological pathways. Moreover, the release of miRNAs into extracellular space in both follicular fluid and blood plasma was not affected by superstimulation treatment.
